# Immobilized Lipase in the Synthesis of High Purity Medium Chain Diacylglycerols Using a Bubble Column Reactor: Characterization and Application

**DOI:** 10.3389/fbioe.2020.00466

**Published:** 2020-05-19

**Authors:** Jiazi Chen, Wan Jun Lee, Chaoying Qiu, Shaolin Wang, Guanghui Li, Yong Wang

**Affiliations:** JNU-UPM International Joint Laboratory on Plant Oil Processing and Safety, Department of Food Science and Engineering, Jinan University, Guangzhou, China

**Keywords:** immobilized lipase, *Candida antarctica* lipase B, medium chain fatty acid, diacylglycerol, purification, water-in-oil emulsion, dioctanoylglycerol, didecanoylglycerol

## Abstract

Novozym^®^ 435, an immobilized lipase from *Candida antarctica* B. (CALB), was used as a biocatalyst for the synthesis of high purity medium chain diacylglycerol (MCD) in a bubble column reactor. In this work, the properties of the MCD produced were characterized followed by determining its practical application as an emulsifier in water-in-oil (W/O) emulsion. Two types of MCDs, namely, dicaprylin (C_8_-DAG) and dicaprin (C_10_-DAG), were prepared through enzymatic esterification using the following conditions: 5% Novozym^®^ 435, 2.5% deionized water, 60°C for 30 min followed by purification. A single-step molecular distillation (MD) (100–140°C, 0.1 Pa, 300 rpm) was performed and comparison was made to that of a double-step purification with MD followed by silica gel column chromatography technique (MD + SGCC). Crude C_8_-DAG and C_10_-DAG with DAG concentration of 41 and 44%, respectively, were obtained via the immobilized enzyme catalyzing reaction. Post-purification via MD, the concentrations of C_8_-DAG and C_10_-DAG were increased to 80 and 83%, respectively. Both MCDs had purity of 99% after the MD + SGCC purification step. Although Novozym^®^ 435 is a non-specific lipase, higher ratios of 1,3-DAG to 1,2-DAG were acquired. Via MD, the ratios of 1,3-DAG to 1,2-DAG in C_8_-DAG and C_10_-DAG were 5.8:1 and 7.3:1, respectively. MCDs that were purified using MD + SGCC were found to contain 1,3-DAG to 1,2-DAG ratios of 8.8:1 and 9.8:1 in C_8_-DAG and C_10_-DAG, respectively. The crystallization and melting peaks were shifted to higher temperature regions as the purity of the MCD was increased. Dense needle-like crystals were observed in MCDs with high purities. Addition of 5% C_8_-DAG and C_10_-DAG as emulsifier together in the presence of 9% of hydrogenated soybean oil produced stable W/O emulsion with particle size of 18 and 10 μm, respectively.

## Introduction

Diacylglycerol (DAG) is made up of two fatty acids esterified to a glycerol backbone. It exists in trace amount in oils of animal and plant sources and exhibits great prospects for industrial application. Approximately 70% of the DAG exists in the form of 1,3-DAG while 1,2-DAG mainly occurs as intermediates from metabolism (Wang et al., 2010). These two isomers differ in terms of the binding positions of the fatty acid acyl groups and hydroxyl groups on the glycerol skeleton and among these isomers, 1,3-DAG is more thermodynamically stable. DAG has been approved by the Food and Drug Association (FDA) as Generally Recognized as Safe (GRAS) food ingredient and it is widely used as emulsifier in various food systems (margarines, mayonnaise, ice-cream, and *etc.*). The acclaimed health benefits of DAG include the suppression of body fat accumulation, lowering the postprandial serum triacylglycerol, cholesterol and glucose level, and improving bone health (Lee et al., 2019). Attributed to the DAG structure, it can be digested and absorbed via different metabolic pathways compared to the triacylglycerol (TAG). Due to the limited amount of DAG that can be extracted from natural sources, DAG can be synthesized via two major pathways, i.e., chemical and enzymatic methods. Although chemical method is often being employed in the industry, this process involves the use of high reaction temperatures at 220–260°C. This is a major drawback as it causes degradation of the thermosensitive polyunsaturated fatty acids, lipid oxidation, and development of undesirable odor, rancidity and color change in the end product. Besides, the use of chemical catalyst is reported to be environmental unfriendly (Xu, 2000). Therefore, synthesis of DAG through the enzymatic pathway has started to gain popularity for its mild reaction temperatures and low energy consumption (Liu et al., 2016).

Lipases acquired from *Candida antarctica* B. (CALB), *Rhizomucor miehei* (RM IM) and *Thermomyces lanuginosus* (TL IM) are widely employed as biocatalysts during the enzymatic synthesis of DAG. The CALB enzyme is among the most stable commercialized lipases as reviewed by Ortiz et al. (2019). Novozym^®^ 435 is produced by Novozymes which is a CALB lipase immobilized on a hydrophobic carrier (acrylic resin). The preparation of DAG using this particular immobilized enzyme through the reaction pathways of glycerolysis (Wang et al., 2018; Zhong et al., 2018), esterification (Liu et al., 2016; Zhang et al., 2017) and hydrolysis (Babicz et al., 2010) has been reported in the literature. Among these reaction pathways, preparation of DAG by enzymatic esterification is of interest in this work due to the limitations of other reaction pathways, i.e., the excess hydrolysis in TAG during hydrolysis reaction and the adsorption of glycerol on the carrier surface of immobilized lipase which limits the contact between lipase and oil phase during glycerolysis (Liu et al., 2016). Some of the more current CALB catalyzed esterification of DAG has been reviewed by Phuah et al. (2015). Most of the reported reactions involved the use of organic solvent as often, solvent is needed to increase the reaction rate and to lower the viscosity of the mixture consisting of fatty acids and glycerol which both are immiscible.

Medium chain fatty acid (MCFA) generally consists of fatty acids of 6–10 carbons such as the caprylic acid (C_8_) and capric acid (C_10_). MCFA is a functional lipid that shows several nutritional and physiological functions especially in the prevention and treatment of metabolic syndromes such as obesity, lipid metabolism, diabetes and hypertension (Nagao and Yanagita, 2010). MCD is a functional lipid that has beneficial effects in the management of obesity and the improvement of lipid metabolism for DAG is less likely to be stored in the adipose tissue. Sek et al. (2002) compared the digestion profiles of long and medium chain TAG, mixture of C_8_/C_10_ MAG- DAG, and long chain TAG (soybean oil and mixture of C_18_ MAG-DAG). It was found that the digestion of MAG/DAG mixtures (C_8_/C_10_ and C_18_) was more rapid than TAG. In the small intestine, MCFA does not form chylomicrons which resulted in energy expenditure through beta-oxidation as reported by Kim et al. (2017) whereby C_8_,_10_-DAG enriched oil reduced body fat mass by stimulating lipolysis in white adipose tissue and thermogenesis in brown adipose tissue. In terms of application, medium chain length (C_8_, C_10_) mono-, di- and triacylglycerols have the potential to be used in pharmaceutical formulation development of poorly soluble compounds to increase their oral bioavailability (Fliszar et al., 2006). The C_8_,_10_-DAG also showed promising results as a delivery system to promote the cutaneous delivery of lycopene and improve the antioxidant activity in the skin (Lopes et al., 2010).

The preparation of high-purity medium chain diacylglycerol (MCD) has great market potential and broad application prospects. Production of structured DAG from MCFAs using biocatalysts is rather a new direction in the field of structured lipids and oil industry. DAG rich in MCD and medium-long chain DAG (MLCD) were prepared by Li et al. (2018) obtaining 39.3% of MCD and 47.3% of MLCD. Das and Ghosh (2019) compared two synthesis ways to prepare MCD (C_8_-DAG) using enzyme and chemical catalysts both under solvent-free conditions using a stirred tank reactor and obtained DAG yields of 58 and 53% when catalyzed by RM IM and Novozym^®^ 435, respectively. However, it has been reported that the preparation of DAG via esterification using mechanical stirring were not encouraged attributed to the viscous and immiscible state of substrate mixtures (Baeza-Jiménez et al., 2013). This could be a contributing factor for the long reaction time (RM IM, 18 h and Novozym^®^ 435, 6 h) needed for the esterification synthesis of C_8_-DAG by Das and Ghosh (2019). Utilization of a bubble column reactor (BCR) has been introduced by Hilterhaus et al. (2008) which offered advantages over the conventional stirred tank reactor. Esterification of viscous reactants in a solvent free system can be performed while being able to prevent damage of the enzyme that was caused by mechanical stirring through gas bubbling technique. There is still a lack of literature reporting on the application of BCR for preparing C_8_ and C_10_ followed by purification and characterization of the DAGs.

This work aims to prepare dioctanoylglycerol (C_8_-DAG) and didecanoylglycerol (C_10_-DAG) via enzymatic esterification catalyzed by immobilized lipase and to obtain high DAG yield under short reaction time. Single-step purification using molecular distillation (MD) and double-step purification using MD followed by silica gel column chromatography (SGCC) was performed to increase DAG yield. Characterization of the MCD in terms of acyl compositions, thermodynamic properties and microscopic structures were performed and the potential application of the MCD that was prepared using the immobilized enzyme as an emulsifier in water-in-oil (W/O) emulsion was determined.

## Materials and Methods

### Materials

Caprylic acid, C_8_ (purity ≥ 99%), capric acid, C_10_ (≥ 99%), and glycerol (≥ 99%) were purchased from Tianjin Chemical Reagent, Co., Ltd. (Tianjin, China). Novozym^®^ 435 (immobilized *Candida antarctica* lipase B) was obtained from Novozymes (China) Biotechnology (Liaoning, China). Emulsifiers such as monoglyceride citrate (CITREM) was obtained from Danisco A/S (Brabrand, Denmark), Span 80 was obtained from Sigma (St. Louis, MO, United States) and polyglycerol polyricinoleate (PGPR) was obtained from Palsgaard Industry A/S (Juelsminde, Denmark). Rapeseed oil (acid value < 0.2%) and fully hydrogenated soybean fat (HSF) were purchased from the local market and Bunge (New York, NY, United States), respectively. Hexane, acetone, petroleum ether, diethyl ether and ethyl acetate (Tianjin Chemical Reagent, Co., Ltd., Tianjin, China) were of analytical grade.

### Enzymatic Synthesis of MCD by Bubble Column Reactor

**FIGURE 1:**
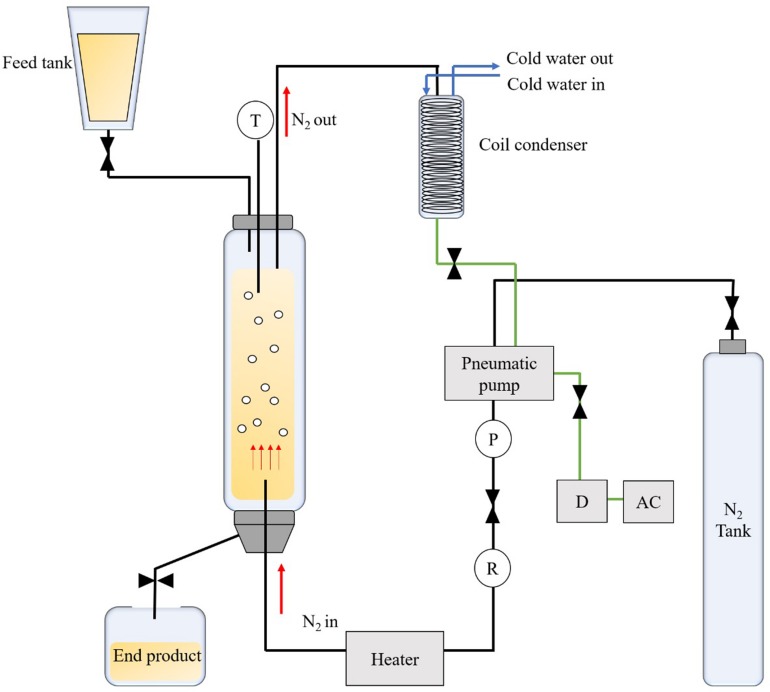
Schematic diagram of a bubble column reactor used for the enzymatic synthesis of medium chain diacylglycerol.

Fatty acids (C_8_, C_10_) of 69 g and glycerol of 331 g, corresponding to a molar ratio of 1:7.5, were added into a BCR (Foshan Handway Technology, Co., Ltd., Guangdong, China) followed by the addition of 5% (w/w) of Novozym^®^ 435 and 2.5% of deionized water. Selection of the reaction conditions was based on preliminary tests (results not shown) and reference to methods and parameters reported by Liu et al. (2016), whereby the optimal reaction conditions were adapted. Figure 1 shows the schematic diagram of the BCR. During the reaction, nitrogen was bubbled up to the reactor (50.0 cm *l* x 5.0 cm *i.d.*) that was filled with substrate and enzyme at a constant flow rate of 10.6 cm/min. The synthesis was carried out at 60°C for 30 min. Nitrogen was recirculated in the system to minimize the consumption using a pneumatic pump. After the reaction was completed, the product was centrifuged at 5000 rpm for 15 min using a high-speed centrifuge (Legend Micro 17R, Guangzhou Bio-Key Technology, Co., Ltd., China) and was allowed to stand for 10–20 min for phase separation. DAG (upper layer) was collected for analysis and the excess glycerol (bottom layer) was discarded.

### Purification

#### Molecular Distillation

Crude MCD product obtained from Section “Enzymatic Synthesis of MCD by Bubble Column Reactor” was purified using a molecular distillator (MD-80, Guangzhou Hanwei Instrument Equipment, Co., Ltd., Guangzhou, China). Purification of MCD was performed at 0.1 Pa, wiped film speed of 300 rpm, and at three different temperatures of 100, 120, and 140°C. Heavy phase (DAG and TAG) and light phase (FFA and MAG) obtained after purification were collected and analyzed.

#### Silica Gel Column Chromatography

A second-step purification using SGCC was performed to increase the purity of the MCD obtained after MD. A preliminary experiment was conducted to determine the suitable type of solvent for elution; petroleum ether: diethyl ether (4:1) and petroleum ether: ethyl acetate (4:1). Mixture of petroleum ether: ethyl acetate was then selected as the eluent/mobile phase for the SGCC purification process due to the separation and purification was more efficient as shown in Supplementary Figure S1.

### Composition of MCD

The MCD composition was analyzed using the method from Wang et al. (2009). A 50.0 mg sample was dissolved in 5.0 mL of *n*-hexane and the solution was filtered through a 0.45 μm filter membrane. The analysis was performed using Agilent gas chromatography system (GC-7820A, Agilent, Santa Clara, CA, United States), equipped with a flame ionization detector (FID) detector and a DB-1ht capillary column (15 m × 0.25 mm, 0.1 μm) (Agilent, Santa Clara, CA, United States). The column pressure was maintained at 20.0 psi and temperature of 380°C. Samples were injected at a volume 0.5 μm with a split ratio of 20:1. The initial oven temperature was set at 50°C for 1 min and increased to 100°C at 50°C/min. The temperature was then raised to 220°C at 80°C/min and to 290°C at 30°C/min. Finally, the temperature was raised to 330°C at 50°C/min for 2 min, and to 380°C at 50°C/min for 3 min.

### DAG Isomers Ratio by NMR

The method of Laszlo et al. (2008) with some modifications was used to determine the ratio of 1,3-DAG to 1,2-DAG isomers using nuclear magnetic resonance spectrophotometer (NM-2, Shanghai Niumai Electronic Technology, Co., Ltd., Shanghai, China). Approximately 10 to 20 mg of purified DAG sample was dissolved in 0.5 mL deuterated chloroform (CDCl_3_) and was then loaded into a nuclear magnetic tube for testing. The conditions for the spectra detection was set at 25°C, pulse width of 12.76 μs, spectral width of 10330.6 Hz, detection frequency 500 MHz, acquisition time of 3.1720 s, and a total of 80 scans.

### Thermodynamic Analysis

Thermodynamic analysis was conducted on a thermogravimetric analyzer (DSC-1, Mettler Toledo, Switzerland). A sample weight of 6 to 10 mg was placed in aluminum pan and was heated according to the following temperature settings and under nitrogen atmosphere at a flow rate of 45 mL/min to obtain a crystallization curve. The temperature program was set as follows: initial temperature of 20°C and was heated to 80°C at 40°C/min and held for 5 min, followed by cooling at −5°C/min to −40°C and held for 5 min. The sample was further heated to 80°C at a rate of 5°C/min to obtain a melting curve. Thermodynamic properties were obtained by analyzing the melting and crystallization curves.

### Crystal Morphology

Samples of purified MCD were heated at 80°C for 5 min and were then cooled to −5°C at 5°C/min. A polarized light microscope (Zeiss Axiovert 200M light microscope, Carl Zeiss Jena, Germany) was used to observe the crystal morphology of the purified MCD.

### Surface Morphology

The surface morphologies of purified MCD samples were observed with a scanning electron microscope (SEM; Nova NanoSEM 430, FEI, Netherlands). Sample (15 to 30 mg) was placed on a clean sample holder under a temperature range of −10 to 10°C, chamber vapor pressure of 7.00 × 10^2^ Pa and a relative humidity of 100% with acceleration voltage at 20 kV.

### Preparation of W/O Emulsion

Although MCD acquired from the double-step purification steps (MD followed by SGCC) were of higher purity, by taking into account the lower yield and higher production cost for the MD + SGCC samples, DAG W/O emulsion was prepared using the MCD obtained from the single-step MD purification. Emulsion formulation includes 80% v/v canola oil, 5% emulsifier (C_8_-DAG or C_10_-DAG), 20% v/v of deionized water and HSF of different amounts (1, 3, 5, 7, and 9% v/v). The oil phase and emulsifier were mixed at 500 rpm for 2 min using a Polytron homogenizer (PT2500E, Kinematica, Switzerland) and aqueous phase was then added into the oil phase drop-wise followed by mixing at 10000 rpm for 5 min. Emulsion was kept at 4°C and analysis were carried out within the next 24 h.

### Emulsion Crystal Morphology and Particle Size Distribution

The crystal morphology was observed using the E-SEM as described in Section “Surface Morphology.” Particle size distribution of emulsion was determined according to the method of Ghosh et al. (2011) using a nuclear magnetic resonance (NMR) analyzer (mq20, bruker, Bremer, Germany). The emulsion droplet size distribution was obtained using the s v5.2 revision 4a version software at a pulse gradient separation of 210 ms and a pulse width of 8.

### Statistical Analysis

All experiments were performed in triplicate. Data were analyzed by analysis of variance (ANOVA) and the mean was compared at the 95% significance level using the Duncan multiple range test (*p* < 0.05) using Origin 8.5 software and SPSS 16.0 software (IBM, United States).

## Results and Discussion

### Enzymatic Synthesis and Purification of MCD

The composition of C_8_-DAG and C_10_-DAG obtained by the immobilized lipase catalyzed esterification (crude MCD) and after purification, respectively, are shown in Table 1. From the reaction, 41.8% of C_8_-DAG and 44.5% of C_10_-DAG were synthesized. The DAG yield obtained was comparatively higher to the results reported in the literature, whereby Das and Ghosh (2019) obtained an approximate C_8_-DAG yield of less than 10% at similar reaction time of 30 min. It can be proposed that the higher DAG yield acquired under short reaction time in this work was ascribed to the usage of a BCR which was more suitable for esterification processes compared to the stirred tank reactor due to the viscous nature of the substrate mixture. The findings in this work were in accordance to the results reported by Liu et al. (2016) in the fast production of long chain DAG using a BCR. Preparation of DAG through the esterification method often involves the formation of water which imparts the reaction. In this work, the ratio of glycerol to fatty acid used was high (7.5:1). The excess glycerol was able to absorb the water formed during esterification and increased the reaction rate (Liu et al., 2011, 2012), obtaining a yield of 41% C_8_-DAG at 30 min. The addition of water at the concentration of 2.5% also aided in activating and maintaining the structure, flexibility and stability of the enzyme (Tao et al., 2013).

**TABLE 1:** Comparison of the compositions of C_8_-DAG and C_10_-DAG before and after purification using molecular distillation.

Substrate	Composition (%)	Crude DAG	Molecular distillation		
			100°C	120°C	140°C
C_8_-DAG	FFA	6.70 ± 0.1	ND	ND	ND
	MAG	46.7 ± 0.1	11.36 ± 0.1^a^	4.66 ± 0.2^b^	2.04 ± 0.1^c^
	DAG	41.8 ± 0.2	77.59 ± 0.2^c^	80.72 ± 0.2^a^	79.39 ± 0.1^b^
	TAG	4.6 ± 0.2	11.05 ± 0.1^c^	14.62 ± 0.1^b^	18.57 ± 0.1^a^
C_10_-DAG	FFA	10.2 ± 0.2	ND	ND	ND
	MAG	39.7 ± 0.1	8.36 ± 0.1^a^	3.25 ± 0.2^b^	2.01 ± 0.2^c^
	DAG	44.5 ± 0.1	80.62 ± 0.2^c^	83.79 ± 0.1^a^	81.73 ± 0.1^b^
	TAG	5.4 ± 0.1	11.02 ± 0.2^c^	12.96 ± 0.2^b^	16.26 ± 0.1^a^

*FFA, free fatty acid; MAG, monoacylglycerol; DAG, diacylglycerol; TAG, triacylglycerol; ND, not detected. All values are means ± standard deviation of triplicate (n = 3); Means that do not share the same letter in the same column are significantly different (p < 0.05).*

The highest DAG yield was obtained from MD performed at 120°C, recording DAG of 80.72 and 83.79% for C_8_-DAG and C_10_-DAG, respectively. When the distillation temperature increased (from 100 to 120°C), the MAG content in MCD reduced significantly while the DAG and TAG content increased. At higher distillation temperature, the boiling point of MCD was achieved and hence higher amount of MCD was carried into the distillate stream. Nonetheless, at higher distillation temperature of 140°C, the concentration of DAG was significantly lower than that at 120°C. Under high temperatures, the DAG underwent esterification to form TAG, as observed with the increment of TAG at 140°C. Yeoh et al. (2014) reported the purification process at higher temperature caused the acyl migration of 1,3-DAG into the thermodynamically unstable intermediate 1,2 (2,3)-DAG which then resynthesized into TAG and MAG. Hence, the best distillation temperature was at 120°C for both C_8_-DAG and C_10_-DAG, obtaining DAG with a purity of 80% (C_8_-80% DAG) and 83% (C_10_-83% DAG), respectively. A second-purification step using SGCC was performed in order to further increase the purity of the MCD. A purity of 99% was attained for both C_8_-DAG (C_8_-99% DAG) and C_10_-DAG (C_10_-99% DAG). The purity increased by a difference of 18.28% and 15.21% for C_8_-DAG and C_10_-DAG, respectively, as both the MAG and TAG were effectively separated from the MCD.

**FIGURE 2:**
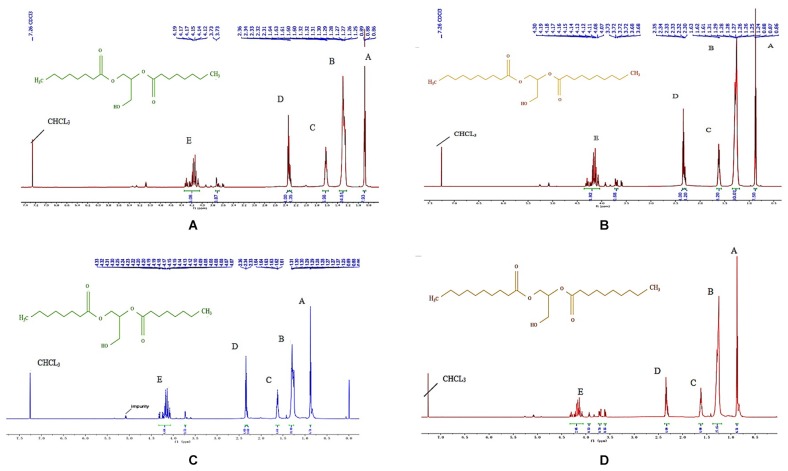
^1^H NMR spectrums of **(A)** 80% C_8_-DAG, **(B)** 83% C_10_-DAG, **(C)** 99% C_8_-DAG, and **(D)** 99% C_10_-DAG and the assignments to the functional groups of molecules are reported in Table 2.

Figure 2 shows the ^1^H NMR spectrums of the one-step purified and double-step purified C_8_-DAG and C_10_-DAG. The respective proton NMR assignments are reported in Table 2. From Figure 2, the hydrogen atom of the -CH_3_ functional group is the most electronegative, so it appears in the position at the down field region. According to the electronegativity of the functional group hydrogen atom and the characteristic peak integration area ratio, the position of each functional group in the product was identified. The multiple peaks in position (E) for both C_8_-DAG and C_10_-DAG corresponded to the presence of 1,2-DAG and 1,3-DAG, hence the region of interest was between 3.5 and 4.5 ppm. The 1,3-DAG was less electronegative than 1,2-DAG due to the differences in terms of their molecular arrangements. The chiral carbon atom of 1,3-DAG was bonded to a hydroxyl group and a hydrogen atom, weakening the electronegativity of the chiral carbon and recorded a chemical shift around 4.2 ppm. 1,2-DAG was more electronegative due to the high electronegativity of hydroxyl groups compared to other carbon atoms, shifting the signal to downfield region with a chemical shift around 3.7 ppm. By integrating the peaks for both MD purified C_8_-DAG and C_10_-DAG (Figures 2A,B), the ratios of 1,3-DAG and 1,2-DAG were 5.8:1 and 7.3:1 respectively. As for the peaks obtained from the DAG samples after the double-purification steps (Figures 2C,D), the ratios of 1,3-DAG and 1,2-DAG were 8.8:1 and 9.8:1, respectively. The higher ratio of 1,3-DAG was attributed to the use of Novozym^®^ 435 which can potentially be 1,3-regioselective. Similar findings has been reported by Duan et al. (2013) whereby high 1,3-diolein yield (93.7%) was obtained in comparison to the yield of 1,2-diolein (2.6%) in the reaction catalyzed by Novozym^®^ 435.

**TABLE 2:** ^1^H NMR assignments of the DAG in purified C_8_-DAG and C_10_-DAG.

Substrate	Peak	Compound	Functional group	δ (ppm)
80% C_8_-DAG	a	C,H	CH_3–_	0.85–0.90
	b	C,H	-(CH_2_)_4–_	1.21–1.38
	c	C,H	-CH_2–_	1.57–1.63
	d	C,H,0	-CH_2_COO-	2.28–2.38
	e	C,H,O	-CH_2_CHOHCH_2_-	3.55–4.35
83% C_10_-DAG	a	C,H	CH_3–_	0.80–0.90
	b	C,H	-(CH_2_)_6–_	1.20–1.38
	c	C,H	-CH_2–_	1.58–1.70
	d	C,H,0	-CH_2_COO-	2.25–2.40
	e	C,H,O	-CH_2_CHOHCH_2_-	3.58–4.40
99% C_8_-DAG	a	C,H	CH_3–_	0.76–0.90
	b	C,H	-(CH_2_)_4–_	1.23–1.33
	c	C,H	-CH_2–_	1.57–1.68
	d	C,H,0	-CH_2_COO-	2.27–2.31
	e	C,H,O	-CH_2_CHOHCH_2_-	3.59–4.30
99% C_10_-DAG	a	C,H	CH_3–_	0.75–0.89
	b	C,H	-(CH_2_)_6–_	1.13–1.36
	c	C,H	-CH_2–_	1.58–1.63
	d	C,H,0	-CH_2_COO-	2.27–2.41
	e	C,H,O	-CH_2_CHOHCH_2_-	3.59–4.30

### Thermodynamic Properties and Micro-Morphology

**FIGURE 3:**
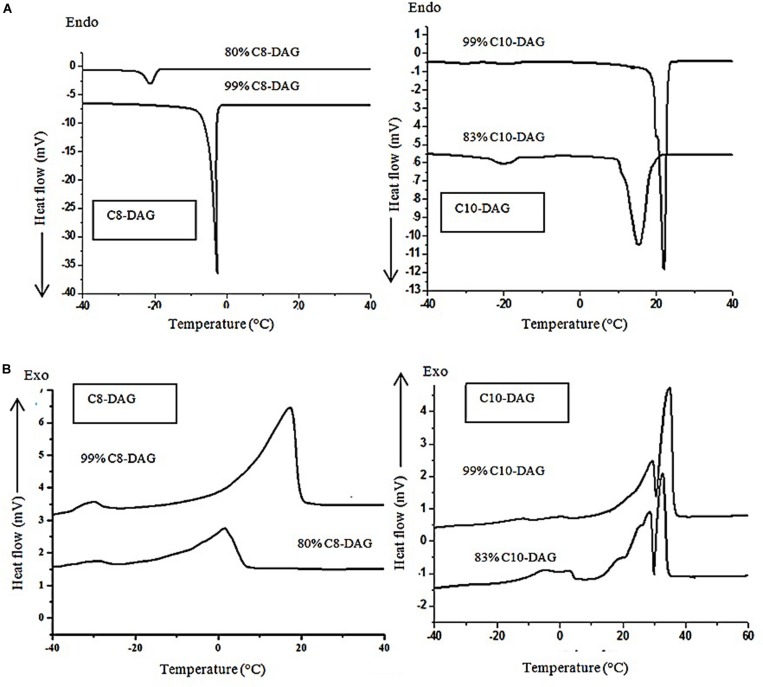
**(A)** Crystallization and **(B)** melting curves of C_8_-DAG and C_10_-DAG after single- and double-step purification.

Figure 3 shows the (A) crystallization and (B) melting curves of 80% C_8_-DAG, 83% C_10_-DAG, 99% C_8_-DAG and 99% C_10_-DAG. The main temperature peaks and the enthalpies of the samples were summarized in Table 3. Both 80% C_8_-DAG and 99% C_8_-DAG showed one crystallization peak at −21.15 and −2.56°C, respectively. Similarly, crystallization peak for 83% C_10_-DAG was at lower temperature than that of 99% C_10_-DAG, recording peaks at 15.55 and 22.12°C, respectively. It was postulated that this phenomenon was attributed to the presence of TAG in the MCD samples of lower purity, causing the reduction in the crystallization temperature as TAG formed crystals by van der Waals force instead of hydrogen bonds (Silva et al., 2015). In terms of the melting curve, the 80% C_8_-DAG showed melting behavior at −23.62°C and was completely melted at 9.76°C while 99% C_8_-DAG showed two melting peaks at −22.64 and 21.21°C. The melting curve of C_10_-DAG showed unresolved peaks (peak II and III in 83% C_10_-DAG, peak I and II in 99% C_10_-DAG), attributed to the transition of unstable crystal to stable crystal form during the melting process. The sharp peak for all high purity (99%) MCD indicated that nucleation and crystal growth were faster, forming large crystal structures (Jansen and Birch, 2009).

**TABLE 3:** Peak temperatures and cooling/melting enthalpies of C_8_-DAG and C_10_-DAG obtained from single- and double-step purification process.

Curve	Sample	Purity of MCD (%)	Peak	Onset (°C)	Offset (°C)	Peak (°C)	Enthalpy (J/g)
Crystallization	C_8_-DAG	80	I	–18.36	–32.18	–21.15	33.90
		99	I	–1.56	–13.13	–2.56	62.95
	C_10_-DAG	83	I	–15.27	–32.12	–20.47	8.53
			II	23.46	–3.89	15.55	86.52
		99	I	23.86	16.43	22.12	92.84
Melting	C_8_-DAG	80	I	–38.08	–23.62	–29.93	1.75
			II	–23.62	9.76	1.64	24.17
		99	I	–40.13	–28.92	–34.65	2.51
			II	–22.64	21.21	12.59	63.39
	C_10_-DAG	83	I	–18.23	5.42	–4.27	14.21
			II	10.89	30.11	28.55	47.96
			III	30.11	35.58	32.67	28.42
		99	I	4.24	40.85	29.26	15.16
			II	30.85	38.24	35.10	46.08

**FIGURE 4:**
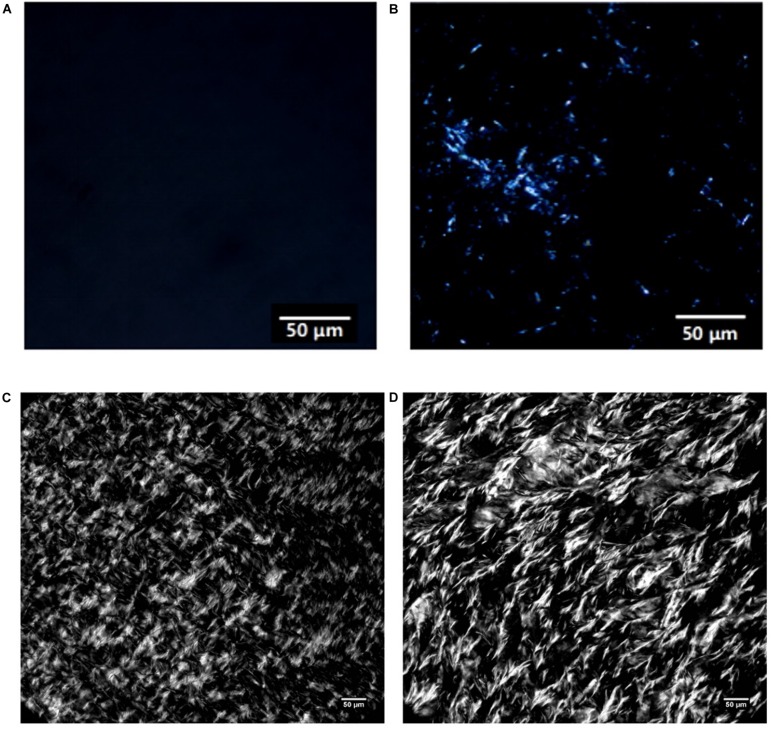
PLM images of medium chain diacylglycerols from single-step purification **(A)** 80% C_8_-DAG and **(B)** 83% C_10_-DAG and from double-step purification **(C)** 99% C_8_-DAG and **(D)** 99% C_10_-DAG.

PLM is widely used for observing the microscopic morphology of crystals in oil systems. Figure 4 shows the PLM images of MCDs obtained from the single- and double-step purification. Two types of polarized microstructures can be observed; the lighter structures represented the crystals while the dark fragments represented the liquid. From Figure 4A, the formation of crystals in the 80% C_8_-DAG was not observed and this was due to the low crystallization temperature of the DAG at −18.36°C as discussed in the previous section. From Figure 4B, fine needle-like crystals were observed with crystal size of less than 30 μm whereas in Figures 4C,D, the crystals were tightly packed together, suggesting the crystallization process occurred through flocculation. DAG normally exists in the form of flocculent crystal which was made up of mainly β-crystals. Studies have shown that the formation of β-crystal started from fine particles and when it reached a certain stage, they started to form needle-like crystals (Himawan et al., 2006). Overall, the crystal particles in C_8_-DAG were smaller than that of C_10_-DAG. Comparing the crystal morphologies in between the DAG of lower purity (80 and 83%) to that of high purity DAG (99%), all the DAGs showed the formation of needle-like crystals while the crystals in high purity DAG were more compact with larger crystal size.

**FIGURE 5:**
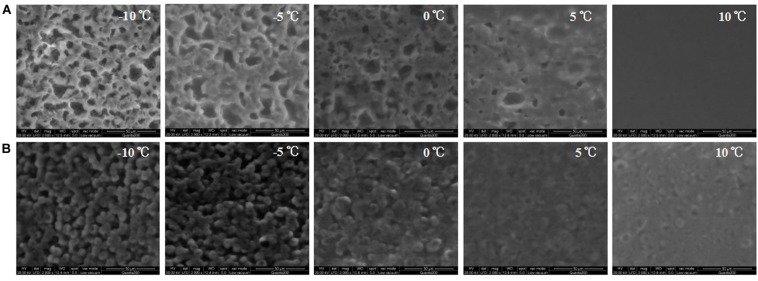
ESEM images of **(A)** C_8_-DAG and **(B)** C_10_-DAG with purity of 99% under different temperatures of –10 to 10°C.

Figure 5 shows the ESEM images of DAG obtained from the double-step purification process. Images of DAGs of lower purity were not shown as both the 80% C_8_-DAG and 83% C_10_-DAG existed in liquid state at −10 to 10°C and hence the surface morphology was not able to be detected using the ESEM. The C_8_-DAG showed irregular crystal network structures with many large voids at −10°C. It was evident that the crystal network of C_8_-DAG gradually loosed its structure into the amorphous state and completely melted when the temperature was increased to 10°C. This observation corresponded to the thermodynamic results obtained whereby the C_8_-DAG had a lower melting point compared to that of C_10_-DAG (Figure 3). Therefore, under similar temperature of 10°C, C_10_-DAG still maintained its network structure whereas the C_8_-DAG had completely melted. On the other hand, the C_10_-DAG showed a crystal network which was of more regular shape with smaller voids at −10°C. Similarly, as the temperature increased, the crystal networks started to dissociated. However, comparing to that of C_8_-DAG, the C_10_-DAG showed the present of cross-linked aggregate structure on the surface at 10°C attributed to the higher melting point of C_10_-DAG.

### Preparation of W/O Emulsion

A preliminary experiment was conducted to determine the effect of different emulsifiers during the preparation of W/O emulsion, i.e., 1% CITREM, 1% SPAN 80, and 1% PGPR. From the emulsion microstructure (Supplementary Figure S2), the use of 1% PGPR produced the most desirable emulsion (water droplet size of 3 μm) attributed to its low HLB value and the long carbon chain structure. Hence, in order to achieve the similar effect of emulsification provided by PGPR, 5% of MCD (calculated based on the HLB values) was added during the preparation of emulsion. The HLB value is shown in Supplementary Table S1. It was found that the addition of 5% of DAG failed to stabilize the emulsion and the water droplets were dispersed with a particle size of 10 μm (Supplementary Figure S2). It was proposed that the addition of HSF which has higher melting point was able to stabilize the emulsion system. Hence, the effect of adding HSF at different concentration in the W/O emulsion preparation was determined. The lower purity MCD (80% C_8_-DAG and 83% C_10_-DAG) was used for the preparation of W/O emulsion instead of the MCD with higher purity of 99% by taking the time and cost factor into consideration.

**FIGURE 6:**
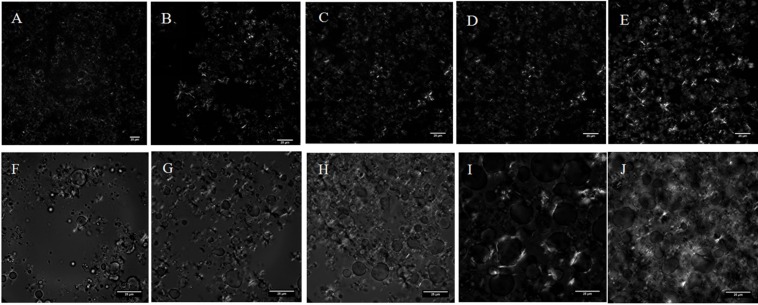
Microscopic images of fat crystals in the emulsion system prepared using a mixture of C_8_-DAG and HSF [**(A)** 5% C_8_-DAG + 1% HSF; **(B)** 5% C_8_-DAG + 3% HSF; **(C)** 5% C_8_-DAG + 5% HSF; **(D)** 5% C_8_-DAG + 7% HSF; **(E)** 5% C_8_-DAG + 9% HSF]; Mixture of C_10_-DAG and HSF [**(F)** 5% C_10_-DAG + 1% HSF; **(G)** 5% C_10_-DAG + 3% HSF; **(H)** 5% C_10_-DAG + 5% HSF; **(I)** 5% C_10_-DAG + 7% HSF; **(J)** 5% C_10_-DAG + 9% HSF].

When only HSF was present in the emulsion system (0% of DAG), there was no occurrence of interfacial crystallization in the emulsion (Supplementary Figure S3) and the crystal was formed mainly in the continuous oil phase. The addition of 5% of DAG in combination with 1 to 9% of HSF showed significant surface activity in the emulsion as shown in Figure 6. The addition of 5% DAG to 1% HSF promoted the surface activity and crystallization occurred at the oil-water interface. This was due to the presence of the hydroxyl group in the DAG carbon chain that extended to the water phase through hydrogen bonding while the acyl chain in HSF-TAG through the van der Waals force, was absorbed into the oil phase. When the interaction between HSF-TAG on the interface continues to accumulate, the DAG mediated fat crystallization at the oil-water interface. Thus, the water droplets in the oil phase were stabilized (Rousseau, 2013). When 3% of HSF was added, the water droplets were covered with formation of crystals; addition of 5% of HSF showed the formation of crystals at the oil-water interface and the presence of some crystals in the continuous phase; addition of 7% of HSF showed the amount of crystals in the continuous phase increased to form a denser crystal network; addition of 9% of HSF showed the continuous phase was substantially filled with crystals and the emulsion structure was denser. Hence, the DAG was able to promote the formation of interfacial crystallization of HSF on the water droplet surface. As the concentration of HSF increased, the interfacial crystallization approached saturation and the crystal networks appeared in the continuous phase with higher number of crystals. The structure becomes denser and the stability of the emulsion was further enhanced.

**FIGURE 7:**
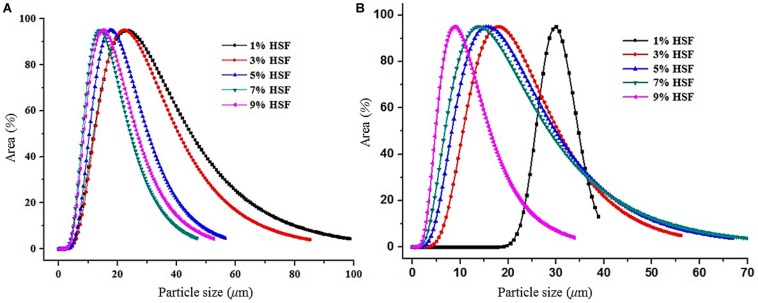
The droplet size of water-in-oil emulsion produced with 5% of **(A)** C_8_-DAG and **(B)** C_10_-DAG in combination with different percentage of hydrogenated soybean fat (HSF).

The emulsion droplet size was analyzed using a pfg-NMR nuclear magnetic resonance spectrometer as shown in Figure 7. The average droplet size of C_8_-DAG and C_10_-DAG added with 1% of HSF were 32 and 35 μm, respectively. As the content of HSF increased, the particle size distribution curve shifted to the left whereby the average droplet size decreased. C_8_-DAG and C_10_-DAG added with 9% of HSF showed an average droplet size of 18 and 10 μm, respectively. The distribution curve was also narrower compared to the lower concentration of HSF, indicating a more uniform distribution of droplet size and hence a more stable emulsion.

## Conclusion

In this work, immobilized lipase Novozym^®^ 435 was successfully employed to catalyze the esterification between glycerol and fatty acids to produce C_8_-DAG (41%) and C_10_-DAG (44%) in a bubble column reactor during a short period of time (30 min). After one-step purification (MD), the purity of MCD was increased to 80–83% and the double-step purification increased the purity to above 99%. Novozym^®^ 435 can be potentially 1,3-regioselective as the concentration of 1,3-DAG produced was significantly higher than that of 1,2-DAG. The thermodynamic properties (melting and crystallization properties) and crystal morphologies of MCDs were altered post-purification. The produced MCD showed great potential to be applied in a W/O emulsion preparation as a stable W/O emulsion was obtained using MCD (C_8_-DAG purity of 80% and C_10_-DAG with purity of 83%) in combination with HSF.

## Data Availability Statement

All datasets generated for this study are included in the article/Supplementary Material.

## Author Contributions

JC carried out the experiment and performed data analysis with the support from SW and GL. WL took the lead and wrote the manuscript with input from all authors. CQ provided insights and supervised the project. YW acquired funding and supervised the project.

## Conflict of Interest

The authors declare that the research was conducted in the absence of any commercial or financial relationships that could be construed as a potential conflict of interest.
