# Supplementary material for: Immobilized Lipase in the Synthesis of High Purity Medium Chain Diacylglycerols Using a Bubble Column Reactor: Characterization and Application
Source: Front Bioeng Biotechnol. 2020 May 19;8:466. doi: 10.3389/fbioe.2020.00466 (PMC7248569; doi:10.3389/fbioe.2020.00466)
Supplement: Supplementary file 1 [file Data_Sheet_1.pdf]

Supplementary Material

Immobilized lipase in the synthesis of high purity medium chain diacylglycerols using a bubble column reactor: Characterization and application

Jiazi Chen[1a], Wan Jun Lee[1a], Chaoying Qiu[1], Shaolin Wang[1], Guanghui Li[1], Yong Wang[1*]

[1] JNU-UPM International Joint Laboratory on Plant Oil Processing and Safety, Department of Food Science and Engineering, Jinan University, Guangzhou 510632, China

[a]*These authors contributed equally to this work and hence should be considered as co-first author*

*** Correspondence:**
Yong Wang
twyong@jnu.edu.cn

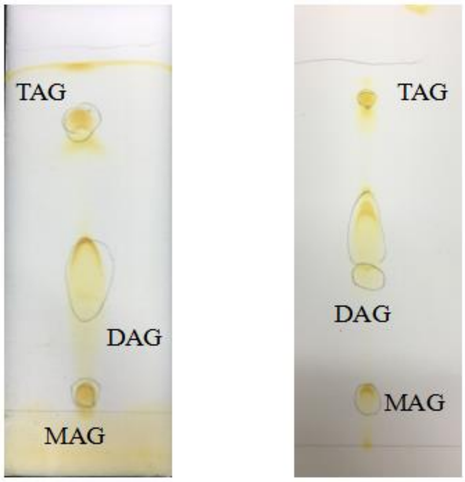

Figure S1 Thin layer chromatograms of different mobile phase systems of (a) petroleum ether: diethyl ether (4:1) and (b) petroleum ether: ethyl acetate (4:1) used to elute and separate the synthesized medium chain diacylglycerols.

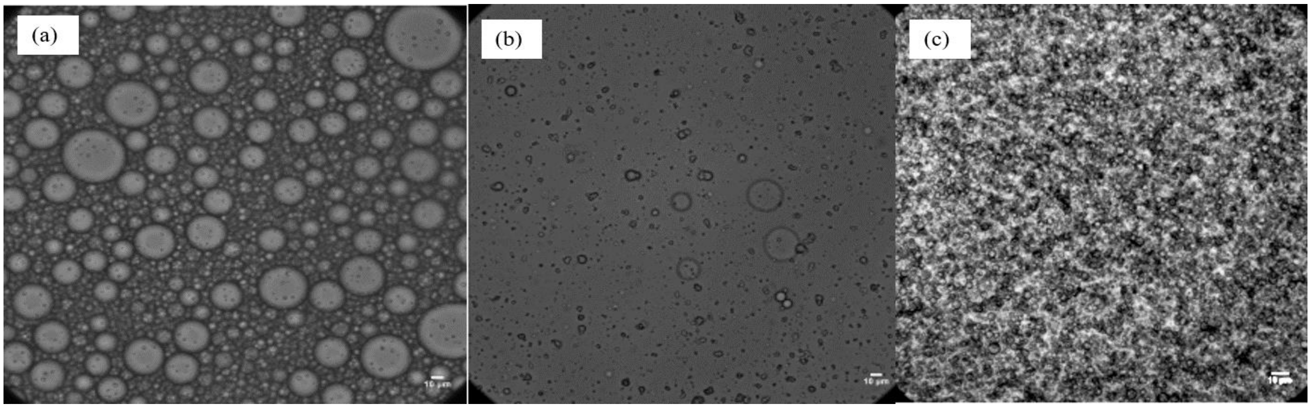

Figure S2 Microscopic images of emulsions prepared using different emulsifiers (a) 1% CITREM, (b) 1% SPAN 80 and (c) 1% PGPR at magnification of 400X.

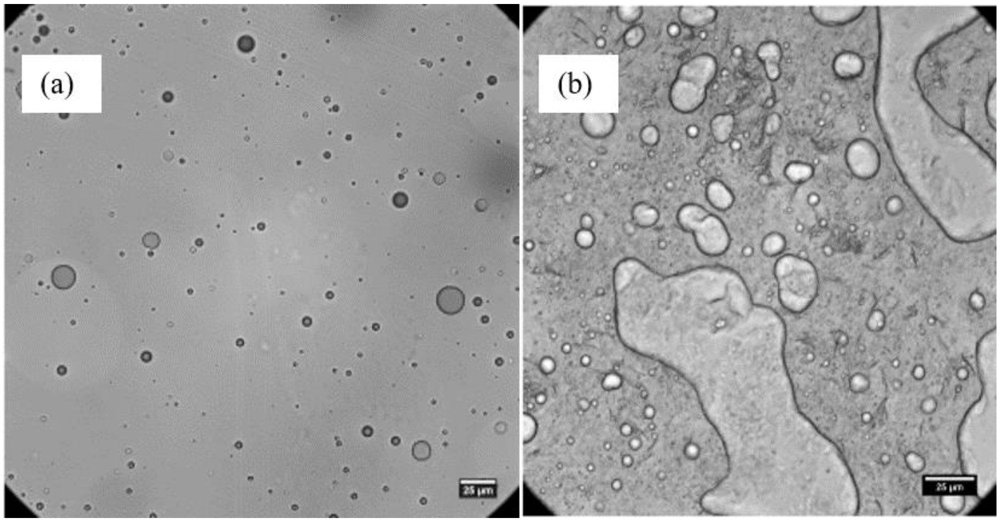

Figure S3 Microscopic images of freshly-prepared emulsion using 5% of (a) C_8-DAG and (b) C_{10}-DAG at a magnification of 640X.

Table S1 Calculated HLB values of the various emulsifiers used

Emulsifier	HLB value
C_8-DAG	4.70
C_{10}-DAG	4.04
CITREM	3-4
SPAN 80	4.3
PGPR	1.5

The HLB values were calculated using the Griffin's method.
